# Structure and Analysis of R1 and R2 Pyocin Receptor-Binding Fibers

**DOI:** 10.3390/v10080427

**Published:** 2018-08-14

**Authors:** Sergey A. Buth, Mikhail M. Shneider, Dean Scholl, Petr G. Leiman

**Affiliations:** 1 Institute of Physics of Biologic Systems, École Polytechnique Fédérale de Lausanne (EPFL), BSP-415, 1015 Lausanne, Switzerland; sebuth@utmb.edu (S.A.B.); mm_shn@mail.ru (M.M.S.); 2 Shemyakin Ovchinnikov Institute of Bioorganic Chemistry, 16/10 Mikluho Maklaya Str., Moscow 117997, Russia; 3 Pylum Biosciences, 385 Oyster Point Blvd., Suite 6A, South San Francisco, CA 94080, USA; dean@avidbiotics.com

**Keywords:** R-type pyocin, bacteriocin, contractile injection systems, *Pseudomonas aeruginosa*, X-ray crystallography, receptor-binding protein

## Abstract

The R-type pyocins are high-molecular weight bacteriocins produced by some strains of *Pseudomonas aeruginosa* to specifically kill other strains of the same species. Structurally, the R-type pyocins are similar to “simple” contractile tails, such as those of phage P2 and Mu. The pyocin recognizes and binds to its target with the help of fibers that emanate from the baseplate structure at one end of the particle. Subsequently, the pyocin contracts its sheath and drives the rigid tube through the host cell envelope. This causes depolarization of the cytoplasmic membrane and cell death. The host cell surface-binding fiber is ~340 Å-long and is attached to the baseplate with its N-terminal domain. Here, we report the crystal structures of C-terminal fragments of the R1 and R2 pyocin fibers that comprise the distal, receptor-binding part of the protein. Both proteins are ~240 Å-long homotrimers in which slender rod-like domains are interspersed with more globular domains—two tandem knob domains in the N-terminal part of the fragment and a lectin-like domain at its C-terminus. The putative substrate binding sites are separated by about 100 Å, suggesting that binding of the fiber to the cell surface causes the fiber to adopt a certain orientation relative to the baseplate and this then triggers sheath contraction.

## 1. Introduction

The chromosomes of many *Pseudomonas* species carry a cluster of genes for one or both of the two types of high-molecular-weight pyocins, the R type and the F type [1]. For example, the “laboratory” strain *P. aeruginosa* PAO1 contains a cluster of both pyocins (the R upstream of the F, genes *pa0610*-*pa0648*) between the *trpE* and *trpG* genes of its tryptophan operon [1]. The cluster is controlled by a common 5’-end regulatory element comprising the activator PrtN (PA0610) and its repressor PrtR (PA0611) [2]. Production of pyocins is triggered by UV irradiation or mitomycin C treatment that cause activation of RecA, which cleaves the repressor, PrtR, allowing the positive regulator, PrtN, to initiate transcription.

Morphologically and functionally, the R-type pyocins resemble the contractile tails of *Myoviridae* bacteriophages [3,4,5,6]. These systems recognize the target cell with the help of fibers or, more generally, receptor-binding proteins that emanate from the baseplate of the particle. Attachment of the fibers to the target cell surface causes structural changes in the baseplate that, in turn, trigger contraction of the external sheath, which drives the internal rigid tube through the host cell envelope. As the pyocins have no capsid, the cell’s cytoplasm becomes open to the external milieu, which causes uncontrollable leakage of ions, destroys the membrane potential, and results in cell death [7]. Mass-spectrometry and bioinformatics show that the mature particle contains 12 proteins that are orthologous to those comprising the conserved core part of the phage T4 tail (Table S1) [8,9].

The killing mechanism of the pyocins is not specific to *Pseudomonas*, and their spectrum is fully determined by the fibers [10,11,12]. The N-terminal part of the fiber attaches it to the baseplate whereas the rest of its structure participates in target cell recognition. The latter can be replaced with a receptor-binding protein from an *Escherichia coli*, *Salmonella enterica* or, *Yersinia pestis* bacteriophage, resulting in a chimerical pyocin particle with a killing spectrum that is the same or wider than that of the donor phage [10,11,12]. The folding of phage and pyocin fibers and, in some cases, their attachment to the particle are controlled by chaperones, which are often encoded by a gene immediately downstream from the fiber gene [13]. In the case of the pyocin with chimerical fibers, both the donor fiber chaperone gene (if present) and the cognate pyocin fiber chaperone gene are required for particle assembly [10].

Five R-type pyocins, called R1 to R5, each with a unique killing spectrum, have been described [14,15]. Their spectra can be represented by a “spectrum tree” with two branches in which R5 is at the root, R1 is one branch, and R2, R4, and R3 form another branch, in that order [16]. The phylogenetic tree of their fiber sequences is roughly similar and contains two branches—R1- and R2-type (Figure S1). The amino acid sequences of the fiber proteins of all the five subtypes are nearly identical from the N-terminus through about one half of the protein. The second half contains stretches of 100% sequence identity and completely dissimilar regions [10], and averages to have slightly greater than 50% identity. Interestingly, the chaperones of these fibers display a significantly greater sequence diversity [10]. It was shown that the l-Rha residue and two distinct d-Glc residues of the outer core of the *P. aeruginosa* lipopolysaccharide (LPS) are part of the receptor sites for R1-, R2-, and R5-pyocins, respectively [17].

The process of host cell recognition and attachment by a bacteriophage or pyocin remains poorly understood. The initial and reversible interaction of receptor-binding proteins with the host cell surface is somehow converted into an irreversible attachment of the particle to the host [18]. It is clear, however, that the overall conformation of receptor-binding proteins changes little upon ligand binding even in proteins that display an enzymatic activity towards cell surface polysaccharides [19,20,21]. Instead, changes in the supramolecular conformation, such as reorientation or other types of global movement of receptor-binding proteins, relative to the rest of the particle appear to initiate the cascade of structural transformations that commit the particle to irreversible host cell binding [22,23].

Here, we present the crystal structures of the R1 and R2 pyocin fiber fragments comprising about two thirds of the fiber and lacking the particle-binding N-terminal domain. These structures represent some of the most complete atomic models of fibrous proteins ever studied in tailed phages or pyocins [8,24,25,26,27,28]. We found that both R1 and R2 pyocin fiber fragments form a ~240 Å-long homotrimer that contains a rod-like and a shaft-like domain, two tandem knob domains, and the C-terminal lectin-like domain. The most diverse regions of the fiber amino acid sequence, which likely determine the killing spectrum, map onto the second knob and C-terminal domains, which are about 100 Å away from each other. Such a distribution of binding sites makes it possible to orient the fiber relative to the target cell surface and fix it in an orientation that will lead to subsequent conformational changes in the baseplate.

## 2. Materials and Methods

### 2.1. Engineering and Choice of the Construct for Expression

The R2 pyocin fiber and chaperone genes (*pa0620* and *pa0621* of *P. aeruginosa* PAO1, respectively) were cloned into two separate and compatible expression vectors, pTSL and pATE, respectively. pTSL (Am^R^) is described elsewhere (NCBI database accession number KU314761) [8]. pATE (Cm^R^) is a pACYCDuet-1 derivative with a single instance of the multiple cloning site and a Tobacco Etch Virus (TEV) protease cleavage site downstream from a 5′-end His-tag (GenBank database accession number MH593819). The PA0620 fiber was expressed as a downstream fusion to the following construct: an N-terminal His-tag, a SlyD domain (FKBP-type peptidyl-prolyl cis-trans isomerase folding chaperone that was introduced to enhance protein solubility), and a linker containing a TEV protease cleavage site [8]. The PA0621 chaperone was tagless.

The full-length protein was soluble but showed a tendency to aggregation and therefore was unsuitable for structural studies. Treatment of this protein with trypsin resulted in two stable fragments, the compositions of which were analyzed by mass spectrometry. These fragments had intact C-terminus and comprised the residues, 173–691 and 124–691. We called these fragments PA0620d1 and PA0620d2, respectively. Both fragments were cloned into the same plasmid system, expressed, and purified. The PA0620d2 mutant behaved similarly to the full-length protein. The PA0620d1 mutant was soluble and could be crystallized although the crystals diffracted poorly and this direction was eventually abandoned.

It was shown that a pyocin particle with a chimeric fiber that is composed of residues 1–164 of the cognate R2 pyocin fiber and residues 291–669 of the phage P2 fiber is active against *E. coli* C (phage P2 host) [10]. This P2 fiber fragment corresponds to residues 323–691 of the R2 pyocin fiber in the sequence alignment. We cloned this R2 pyocin fiber fragment (which we called PA0620d3) into the pTSL expression system and found that this fragment was soluble when co-expressed with the PA0621 chaperone, could be purified to homogeneity, and gave diffraction quality crystals.

We then cloned a PA0620d3-like fragment of the R1 pyocin fiber PLES_06171 (residues 323–701) and its chaperone PLES_RS03160 from *P. aeruginosa* LESB58 into the same dual plasmid expression system. The R1 fiber fragment was a more difficult protein to work with than PA0620d3, but it was also eventually crystallized and gave reasonably well diffracting crystals.

### 2.2. Protein Expression and Purification

Protein expression was performed in the B834 (DE3) strain of *E. coli* (a methionine auxotroph). The transformed cells were grown at 37 °C in the 2xTY medium (supplemented with ampicillin at a concentration of 200 μg/mL) with aeration by orbital shaking at 200 rpm until the optical density at 600 nm (OD_600_) reached a value of 0.6. The medium was cooled on ice to a temperature of 18–20 °C and the protein expression was induced by an addition of IPTG to a final concentration of 1 mM. After further incubation at 18 °C overnight (approximately 16 h), the cells were harvested by centrifugation at 5180× *g* for 15 min at 4 °C. The cell pellet was resuspended in 1/50th of the original culture volume of a 20 mM Tris-HCl pH 8.0, 300 mM NaCl, 5 mM imidazole buffer. The cells were lysed by sonication with the sample kept on ice. The cell debris was removed by centrifugation at 35,000× *g* for 15 min at 4 °C. The supernatant was loaded onto a Ni^2+^-precharged 5 mL GE HisTrap FF Crude column (GE Healthcare Life Sciences, Chicago, IL, United States) that was equilibrated with a 20 mM Tris-HCl pH 8.0, 300 mM NaCl buffer. The non-specifically bound material was removed by washing the column with a buffer containing 50 mM Tris-HCl pH 8.0, 250 mM NaCl, and 50 mM imidazole. The affinity-bound material was eluted with a buffer containing 20 mM Tris-HCl pH 8.0, 250 mM NaCl, and 200 mM imidazole. Fractions containing the target protein were pooled together and set up at 25 °C for an overnight digestion with the TEV protease at a protease/protein ratio of 1/100 (*w*/*w*). This reaction mixture was simultaneously dialyzed against a 10 mM Tris-HCl pH 8.0, 3 mM DTT, 1.5 mM EDTA buffer, resulting in cleavage of the His-SlyD expression tag. The digested protein was filtered and purified by ion-exchange chromatography using a GE MonoQ 10/100 GL column and 0 to 650 mM NaCl gradient in a 20 mM Tris-HCl pH 8.0 buffer. Relevant fractions were combined and concentrated using Sartorius (Sartorius AG, Göttingen, Germany) ultrafiltration devices with a molecular weight cutoff of 50,000 to a volume of ~5 mL. This sample was loaded onto a GE HiLoad 16/60 Superdex 200 size-exclusion column equilibrated with 10 mM Tris-HCl pH 8.0, 150 mM NaCl. Fractions containing the pure protein were combined and concentrated with the help of a similar Sartorius ultrafiltration unit. Proteins were stored in the same buffer at +4 °C until they were used for crystallization. All purification buffers and the final protein solution contained NaN_3_ at a concentration of 0.02% (*w*/*v*).

To produce a Se-methionine (SeMet) derivative of R2 PA0620d3, the cells were first grown in the 2xTY medium until OD_600_ of 0.3, then pelleted by centrifugation at 3315× *g* for 10 min at 20 °C, and transferred to the SelenoMet Medium (Molecular Dimensions, Newmarket, Suffolk, UK) prepared according to the manufacturer’s instructions and supplemented with ampicillin at a concentration of 200 μg/mL. All the subsequent steps including the expression at low temperature and protein purification were the same as for the native protein.

### 2.3. Crystallization, Data Collection, and Structure Determination

For crystallization, purified R1 and R2 pyocin fiber fragments were concentrated to 9 and 11 mg/mL, respectively. The initial crystallization screening was carried out by the sitting drop method in 96 well 2-lens MRC plates (SWISSCI AG, Neuheim, Switzerland) using JBScreens (Jena Bioscience, Jena, Germany) crystallization screens. Optimization of crystallization conditions was performed in Jena Bioscience SuperClear pregreased 24 well plates by hanging drop vapor diffusion. Crystallization drops of the 24 well plate setup contained 1.5 μL of the protein solution in 10 mM Tris-HCl pH 8.0, 150 mM NaCl mixed with an equal volume of the well solution. Best R1 and R2 fiber crystals were obtained at +18 °C with the protein concentration at 7.4 and 7 mg/mL equilibrated against 750 μL of the well solution containing 4% PEG 4000, 100 mM PIPES pH 6.1, 140 mM Na_2_(C_3_H_2_O_4_) (sodium malonate) and 5% PEG 6000, 100 mM Tris-HCl pH 8.5, 180 mM KCl, respectively. For data collection, the crystals were incubated for 20–45 s in a cryo solution that contained all the well solution components and, additionally, 30% (*v*/*v*) of glycerol for the R1 and 25% (*v*/*v*) of ethylene glycol for the R2 fiber, and flash frozen in a vaporized nitrogen stream at 100 K.

Crystallographic data collection was carried out at the X06SA PXI beam line of the Swiss Light Source (SLS) at the Paul Scherrer Institute (SLS, Villigen, Switzerland). Although best crystals of R2 PA0620d3 diffracted to better than 1.7 Å resolution, the resolution of the collected diffraction data had to be limited to 1.9 Å even when using a PILATUS 6M detector (424 × 435 mm^2^, 2463 × 2527 pixels) because of the excessive spot overlap due to a large unit cell and a relatively high mosaicity (Table 1). The structure was solved by the single-wavelength anomalous diffraction (SAD) technique using a SeMet derivative [29]. The SAD data were collected at a wavelength of the maximum Se adsorption (K-line) that was established with a fluorescent scan. For both proteins (R1 and R2) the native data were collected at a wavelength of 1 Å (Table 1).

The diffraction data was indexed, integrated, and scaled with the program XDS [30,31]. The Se sites were located with the SHELX_CDE program suite [32] using the HKL2MAP interface [33]. The program SOLVE [34] was used for the refinement of sites found by SHELX and for phasing. The SOLVE phases were improved by non-crystallographic symmetry (NCS) averaging and solvent flattening with the program RESOLVE [34]. The program ARP/wARP [35] was used for automated model building. Further refinement of the atomic model was performed with the programs Coot [36], Refmac5 [37], and PHENIX [38] with TLS [39]. Details of data reduction and refinement are given in Table 1.

The structure of the R1 pyocin fiber was solved by the molecular replacement method [40] with the PHASER [41] program as part of the CCP4 program package [42] using the PA0620d3 structure as a search model. Similar to the R2 fiber structure, Coot and PHENIX with TLS were used for the refinement of the atomic model of the R1 fiber.

### 2.4. Molecular Graphics, Analysis of Surface Properties, and Bioinformatics

All molecular graphics figures were prepared with the program UCSF Chimera [43]. The electrostatic potential was calculated with the program APBS [44,45]. The sequence diversity analysis was performed with a non-redundant set of protein sequences that were identified with the help of the Basic Local Alignment Search Tool (BLAST) [46]. The molecular surfaces were calculated with the program MSMS [47] as implemented in UCSF Chimera. Bioinformatic analysis was performed with the web servers BLAST [46], HHpred [48,49], and Phylogeny.fr [50].

### 2.5. Pyocin Fiber Competition Assay

10^6^ CFU/mL of *P. aeruginosa* strain 13s was incubated for 10 min at 37 °C with varying amounts of the R2 fiber fragment PA0620d3 that carried the His-SlyD expression tag. 10^8^ KU/mL of the R2 pyocin that was purified from the *P. aeruginosa* PAO1 strain [10] was then added and incubated for an additional 20 min (KU, a killing unit, is defined as the amount of activity required to kill a single cell; in this case, it corresponds to a single pyocin particle). The cells were then diluted and spotted onto an LB agar plate. See [10] for KU calculation, strains specification, and pyocins purification protocol.

## 3. Results

### 3.1. R1 and R2 Fibers Are Structurally Similar

**Figure 1:**
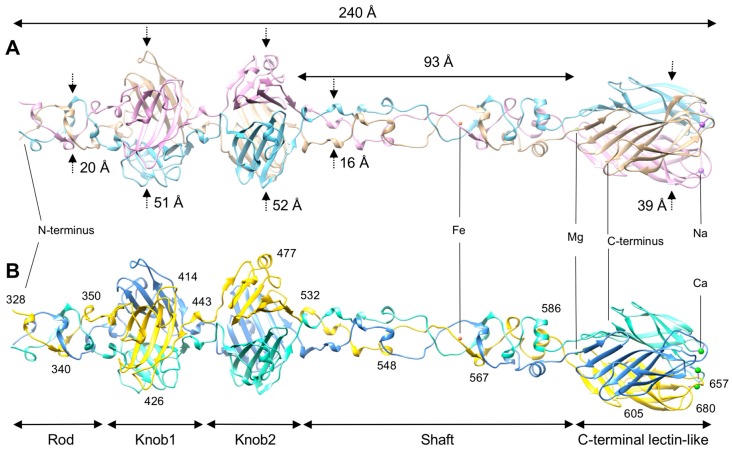
The structure and domain organization of the R1 (panel A) and R2 (panel B) fibers. Domain dimensions, position of the metal ion sites, and residues at strategic positions are indicated. Polypeptide chains are showed in the ribbon representation. Monomers are colored in plum, tan, and sky blue for the R1 molecule (**A**), and gold, cornflower blue, and aquamarine for the R2 molecule (**B**).

The R1 and R2 fiber fragments have a similar domain organization and overall structure (Figure 1). Each protein is a ~240 Å-long fiber that is formed by three intertwined polypeptide chains comprising five domains. Four copies of the “helix-plus-turn” motif create the N-terminal “Rod” domain (residues 328–356). It is followed by two tandem “Knob” domains (Knob1, residues 357–439, and Knob2, residues 445–528) that have similar folds. The two Knobs are followed by a “Shaft” domain (residues 529–598) that is ~93 Å long and contains a buried iron ion approximately in the middle. The remaining C-terminal domain (residues 599–691) is a β-sandwich with a lectin-like fold. About 45% of the total surface area of each monomer is buried in the trimeric interface (32,719 Å^2^ for R1 and 31,680 Å^2^ for R2 fiber). The estimated dissociation energy for R1 and R2 fiber trimers is 196.1 kcal/mol and 192.7 kcal/mol, respectively. This is consistent with the fiber being an SDS-resistant trimer at room temperature as is the case for many other interdigitated fibrous proteins [51,52].

### 3.2. The Rod and Shaft Domains Are Built Using a Helix-Plus-Turn Motif

**Figure 2:**
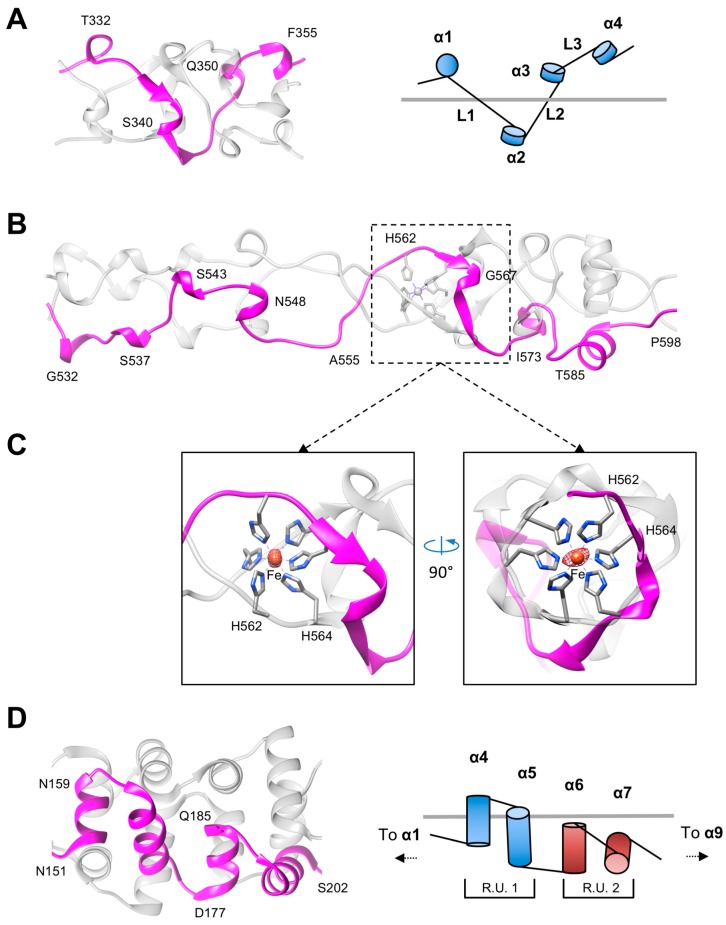
Detailed analysis of the Rod and Shaft domains. The fold and schematic topology overview of the Rod (**A**) and Shaft (**B**) domains. Linkers of variable length connecting the α-helices (α1–α4) are labeled L1, L2, and L3. (**C**) Side view and N-to-C end-on view of the iron-binding motif that is located approximately in the middle of the Shaft domain. The sidechains of histidines constituting the binding site are showed in a stick representation. The centrally positioned iron ion is shown as a rust-colored sphere. The Bijvoet difference Fourier synthesis map is shown as a red mesh and contoured at 4.0 standard deviations above the mean. His-Nε2-Fe coordination bonds are showed with dashed lines. One monomer is colored in magenta and the other two are colored in transparent grey. The R2 fiber model was used for illustration. (**D**) The fold and schematic topology overview of the stem domain fragment of phage phi29 head fiber. R.U. stands for Repeating Unit.

The folds of the Rod and Shaft domains are characterized by a low secondary structure content and complex topology in which the three polypeptide chains extensively interdigitate (Figure 2). Both contain a repeating unit, which we termed the “helix-plus-turn” motif, that consists of a very short α-helix (3–4 residues) followed by a sharp clockwise turn (Figure 2A,B). The motifs are connected by linkers of variable lengths (two to seven residues). The α-helical part of the helix-plus-turn motif is identified as the “niche4r” motif by PDBeMotif [53].

The helix-plus-linker motif is topologically similar to the helix-turn-helix motif of the stem domain of the phage phi29 head fiber Figure 2D [54]. The phi29 stem domain is highly regular: Each turn raises the subsequent α-helix by approximately 10 Å. Additionally, the double-helix repeat units of the super helix region (helices 4–9) related by a 45° turn and a translation of 19.5 Å along the helical axis (Figure 2D). The structure of the pyocin fiber is not as regular because of the varying length of the linkers connecting the helix-plus-turn motifs (Figure 2A,B). Nevertheless, the Shaft and Rod domains have a nearly constant diameter throughout.

Repeats are a common theme in the organization of many fibrous proteins [8,55]. In many cases they can be detected at the amino acid sequence level, such as the heptad repeats in α-helical coiled coils [56] or valine-glycine repeats in β-helices [57,58]. In other cases, where the repeat is short, such as the one described here (Figure 2A,B), it can only be identified at the level of the protein structure. A repeating structure allows for adjustment of the length of the fiber and shows that most fibers are evolving by reusing the same structural element by means of domain or motif duplication events. The function of many fibrous proteins, such as pyocin and phage fibers, involves the binding of cell surface moieties that represent an extremely diverse set of ligands. A structure built with repeats makes it possible to fine tune this binding both spatially and temporarily to the ligand at hand and coordinate it with subsequent conformational changes in the pyocin/phage particle.

### 3.3. R1 and R2 Fibers Contain Buried and Solvent Exposed Metal Ions

**Figure 3:**
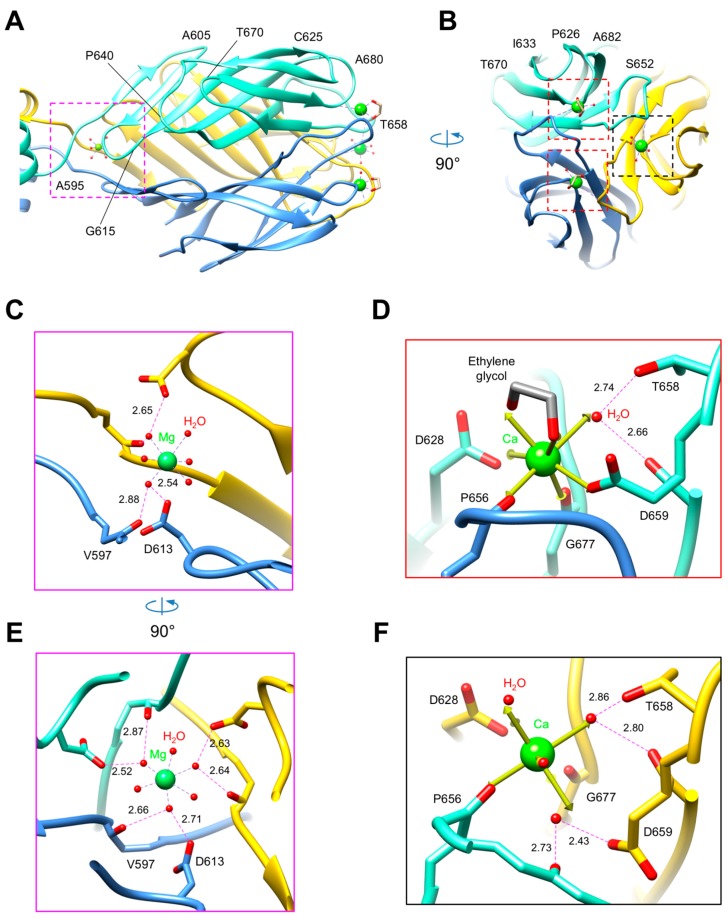
Structure of the R2 fiber C-terminal domain with bound metal ions and ligands. Side view (**A**) and C-to-N end-on view (**B**) of the R2 pyocin receptor binding domain. Cut-away side view (**C**) and C-to-N end-on view (**E**) of the magnesium site. (**D**,**F**) Structure of two different calcium ion binding sites. The three protein chains comprising the trimer are colored in gold, cornflower blue, and aquamarine. The dashed rectangles in the top panels mark the area of detail enlarged in the bottom panels. The magenta rectangle outlines the location of the magnesium ion. The red and black rectangles mark two different calcium-binding sites. The arrows in panel (**C**) represent the ideal coordination geometry for a calcium ion. The hydrogen and coordination bonds are showed as dashed lines. The distances of H-bonds between the outer and inner coordination shells are indicated in Å.

The interior of the Shaft domain in R1 and R2 fibers contains two metal ions—an iron roughly in the middle and a hydrated magnesium in its very C-terminal part where the Shaft transitions into the C-terminal lectin domain. The presence of the iron ion was first detected with X-ray fluorescent spectroscopy. Its location in the structure was then established with the help of the Bijvoet difference Fourier synthesis (Figure 2C). In addition to the buried metal ions, the R1 and R2 fibers contain solvent exposed sodium and calcium ions, respectively, bound to the tip of the C-terminal lectin domain (Figure 3). The identification of these ions is based on the analysis of the coordination geometry, temperature factors of these ions, and those of the surrounding residues, as well as on the correlation with the electron density map. The corresponding statistics are given in Table 2 and Table S2.

The parameters of the magnesium, sodium, and calcium binding sites suggests that these ions are bound to the protein structure in their most common oxidation state (Table 2). The CheckMyMetal web server [59,60] indicates that the valence and therefore the oxidation state of iron ions incorporated into pyocin fibers is (II). As Fe(III) is more stable in solution, it is likely that iron is reduced to Fe(II) after it binds to the protein. The crystallographic refinement statistics for the Fe(II) and Fe(III) ions are nearly identical.

The iron-binding site has an octahedral geometry and is composed of six histidine residues—three symmetry-related copies of His561 and His563 in R1 and His562 and His564 in R2 (Figure 2C). Similar iron-binding sites created by the same HxH motif are found in the structure of the T4 long tail fiber protein gp37 (PDB code 2XGF) [26] and in the central spike proteins of phage P2 (gpV, PDB codes 3QR7 and 3QR8) [52], phage phi92 (gp138, PDB codes 3PQI and 3PQH) [52], and R2 pyocin (PA0616, PDB codes 4S36 and 4S37). Phage T4 short tail fiber protein gp12 also contains an HxH motif, but its crystal structure contains a zinc ion in place of the more common iron [24,25]. However, the purification process of gp12 involved a heat denaturation step and reconstitution in the presence of a zinc salt, so it is unclear whether the zinc ion is the natural ligand of that site in the wild type gp12 protein.

The water shell of the buried hydrated magnesium ion has a nearly perfect octahedral geometry (Figure 3A–C,E, Table 2). The ion is coordinated by three symmetry-related copies of the main chain oxygen of Val596 and side chain of Asp612 in the R1 fiber (Val597 and Asp613 in R2) (Figure 3C,E). The structure of this site is somewhat similar to that of the phage T4 cell-puncturing gp5 protein, where a hydrated magnesium ion is buried in a hydrophobic cavity where it is coordinated by three glutamate residues [61].

The function of the buried iron and magnesium ions is most likely related to protein folding. They might form “reference points” for three nascent protein chains that are about to fold into a trimer [52,62], although there is no experimental data to support this hypothesis at this point. The metal-binding sites could also give the fiber the required balance of stiffness and flexibility because crystal packing forces can elastically bend, but not break, the iron-containing Shaft domain (see Figure S2).

### 3.4. A Small Compound is Buried in the Hydrophobic Interior of the Knob2 and Shaft Domains

The Knob2 and Shaft domains of R1 and R2 fibers display 53% and 74% sequence identity and have a nearly identical main chain traces, which can be superimposed with a root mean square deviation (RMSD) of 0.73 and 0.82Å, respectively. However, their interchain hydrophobic cores display an interesting structural difference. A flat and nearly perfectly triangular density is located on the axis of the Knob2 of the R1 fiber, but no such feature is present in the R2 (Figure S3). A similar triangular density is buried in the N-terminal part of the Shaft of the R2 fiber (residues Phe534-Tyr539), but there is no such “molecule” in the Shaft domain of the R1 fiber (Figure S4). Remarkably, in both cases, the threefold axis of the “molecule” is not parallel to the axis of the trimer and the “molecule” thus does not interact with its threefold environment in a threefold symmetric fashion. The deviation of the “molecule’s” axis from the threefold axis of the fiber is more prominent in the Knob2 domain of the R1 fiber (Figure S3C). All residues of the Knob2 domain cavity in the R2 structure are conserved except Ile491 of R1 is replaced with His491, which gives the cavity a slightly different configuration. In the R2 Shaft domain cavity, the situation is more mysterious as the residues forming the cavity in both fibers are identical (Figure S4G), but the cavity of the R1 fiber is empty.

We attempted to determine the identity of the “molecule” corresponding to the triangular electron density by crystallographic refinement of different compounds possessing a threefold symmetry (nitrate and carbonate ions) or pseudo threefold symmetry (acetate ion and acetone), as well as three water molecules placed in the vertices of the electron density feature. Unfortunately, none of the ligands were a clear favorite because all gave acceptable refinement statistics, agreed with the electron density, and did not distort the surrounding protein residues (Figures S3 and S4). The height of the resulting map peaks, map correlation coefficients, and temperature factors for each refined ligand are given in Table S3. In the structures of R1 and R2 pyocin fibers deposited to the Protein Data Bank, these electron density features are interpreted as water molecules.

### 3.5. Knob-Like Domains Are Found in Saccharide-Binding Tail Fibers and Tailspikes

**Figure 4:**
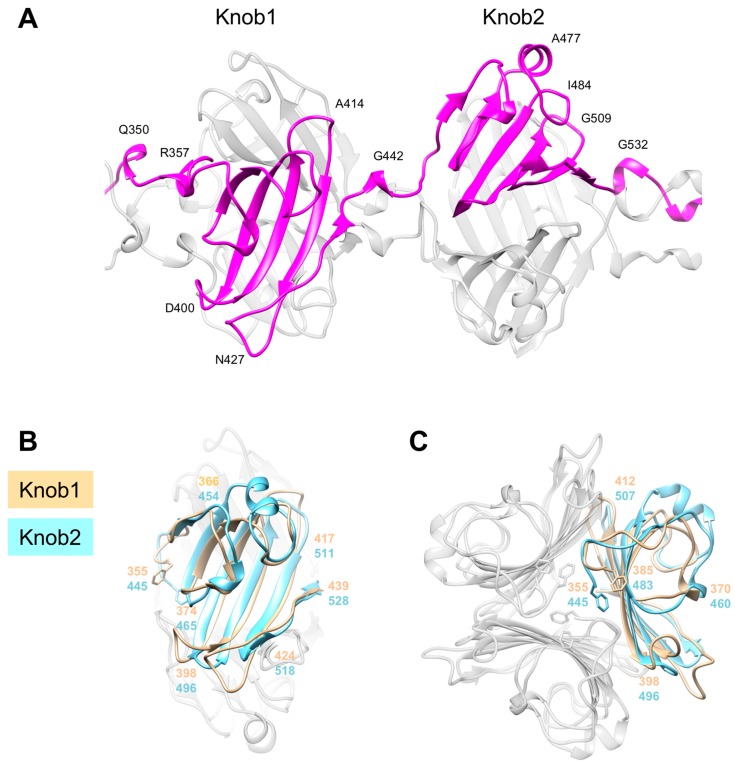
Structure of the two tandem Knob domains Knob1 and Knob2. (**A**) The trace of one of the three chains comprising the trimer. A side view (**B**) and an N-to-C end-on view (**C**) of the superimposed Knob1 and Knob2 domains. Residues are numbered with the color code of the corresponding domain. The R2 fiber model was used in all panels.

The tandem Knob domains, Knob1 and Knob2, have a similar fold that is, essentially, an antiparallel β-sheet with five or six strands (Knob1 and Knob2, respectively) that are connected by loops of variable lengths (Figure 4). One such loop in the Knob2 domain is 16 residues long (S471–R486 in the R2 fiber) and contains a six residue-long α-helix. The loops curve toward the β-sheet and create a partially closed structure. The loops form the outer surface of the molecule whereas the β-sheets are buried in the trimeric interface (Figure 4B). As a consequence, both sides of the β-sheet display hydrophobic side chains. One set of hydrophobic residues points towards the axis of the fiber and mediates interactions between the three chains comprising the trimer. The other, together with the side chains donated by the loops, creates the actual hydrophobic core of the Knob domain (intra-chain interactions). Because of their unusual multi-hydrophobic core structure, Knob-like domains are likely to play an important role in folding of this and other trimeric proteins. The Knob1 and Knob2 domains are connected by a helix-plus-turn motif that is similar to those of the Rod and Shaft domains (Figure 2).

The two Knob domains of the R2 fiber show 20.0% sequence identity on superposition with 201 aligned C_α_ atoms (out of 254) and a root mean square deviation (RMSD) of 2.13 Å (Figure 4B). These domains have clearly evolved from a single ancestor as a result of yet another gene duplication event. Some residues have been retained, but repurposed, in this process. F445 plays a very important role in the structure of Knob2. Its side chain is fully buried and positioned so that it constitutes an integral part of both the inter- and intra-chain hydrophobic cores of the Knob2 domain. The main chain of F445 is exposed to the solution, “sealing” both cores. The equivalent residue in the Knob1 domain is F335. Its side chain is partially exposed to solution, and it is a component of the last helix-plus-turn element (V354–R357) preceding the Knob1 domain.

**Figure 5:**
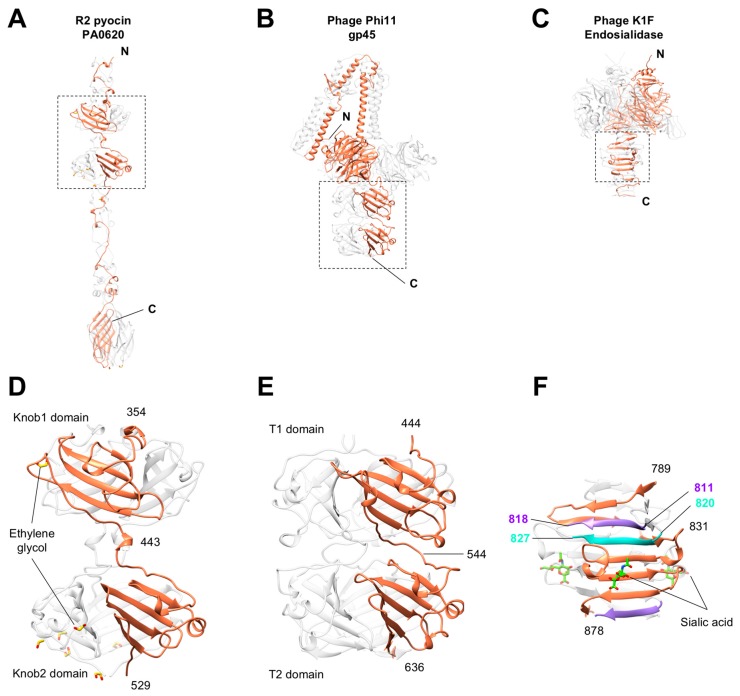
Comparison of the Knob domains with their orthologs from other receptor-binding proteins. Location of the Knob-like domains in the pyocin fiber (**A**), in the putative receptor-binding protein gp45 of phage phi11 [65], and (**B**) in the endosialidase tailspike of phage K1F (where it is called β-prism) [66,67] (**C**). The dashed-line rectangles outline the area of detail shown enlarged in panels (**D**–**F**). Each face of the Knob-like β-prism of the K1F endosialidase (panel **F**) is composed of three polypeptide chains (three different colors). The β-prism forms a smooth extension of the triple-stranded β-helix domain.

A search for protein structures similar to Knob1 and Knob2 domains (performed with DALI [64]) identified similar trimeric domains in other viral proteins, such as the putative receptor-binding proteins gp45 (PDB code 5EFV, Z = 9.8, RMSD = 2.2 Å) [65] and gp144 (PDB code 5M9F, Z = 8.2, RMSD = 2.1 Å) of Staphylococcus phages Phi11 and K, respectively, and T4 proximal long tail fiber protein gp34 (PDB code 5NXF, Z = 7.6, RMSD = 2.5 Å) [27]. In particular, the C-terminal “tower” of the putative receptor-binding protein of *Staphylococcus* phage Phi11 (PDBID 5EFV) features two such domains in tandem, identical to the R-type pyocin fiber organization (Figure 5).

Endosialidase tailspike proteins of phages K1F (PDB code 3JU4) [66,67], and Phi92 (PDB code 4HIZ) [51] as well as the KflA tailspike of K5A (PDB code 2X3H) [68] and gp42 tailspike of *A. baumannii* phage AS12 (PDB code 6EU4, N. I. M. Taylor, M. M. Shneider, P. G. Leiman, unpublished data) all also possess a Knob-like domain. It is called a β-prism in the K1F endosialidase and is located downstream from the sialidase domain (Figure 5B). The Knob-like β-prism domain is further extended by a triple-stranded β-helix. The module comprising the β-prism and the triple-stranded β-helix of endosialidases is, in turn, structurally similar to the C-terminal, membrane-puncturing module of central spike proteins of contractile tail bacteriophages [52,69,70]. In all these proteins, the transition between the Knob-like β-prism and triple-stranded β-helix is very smooth—a ladder-like trace of the polypeptide chain of the Knob-like β-prism domain is continued by the triple-stranded β-helix without interruption. Thus, β-prisms, Knob-like fiber domains and the triple-stranded β-helices are likely to have a common ancestor.

The presence of Knob-like domains in host cell recognition and binding proteins of pyocins and phages is clearly dictated by their conserved function. A Knob-like domain of phage K1F endosialidase was shown to bind a fragment of sialic acid [66,67] (Figure 5C,F), which is the host cell surface molecule that is recognized by K1F during infection [71]. Knob-like domains are likely to be involved in host cell recognition and binding in other pyocin/phage systems.

### 3.6. The C-Terminal β-Sandwich Domain Has a Lectin-Like Fold with a Negatively Charged Surface Groove

Residues 596–691 and 595–701 comprise the C-terminal β-sandwich domain of the R2 and R1 fibers, respectively. Six of the eight β-strands of the β-sandwich form a jellyroll fold-like structure giving rise to an asymmetric sandwich with five and three strands per “side” (Figure 3A). The folds of the R1 and R2 fiber domains are similar and the two structures can be superimposed with an RMSD of 1.35 Å between 249 equivalent Cα atoms in the alignment (out of 318 atoms in R1 and 285 in R2) (Figure S5). The sequence identity of this superposition is 40%.

**Figure 6:**
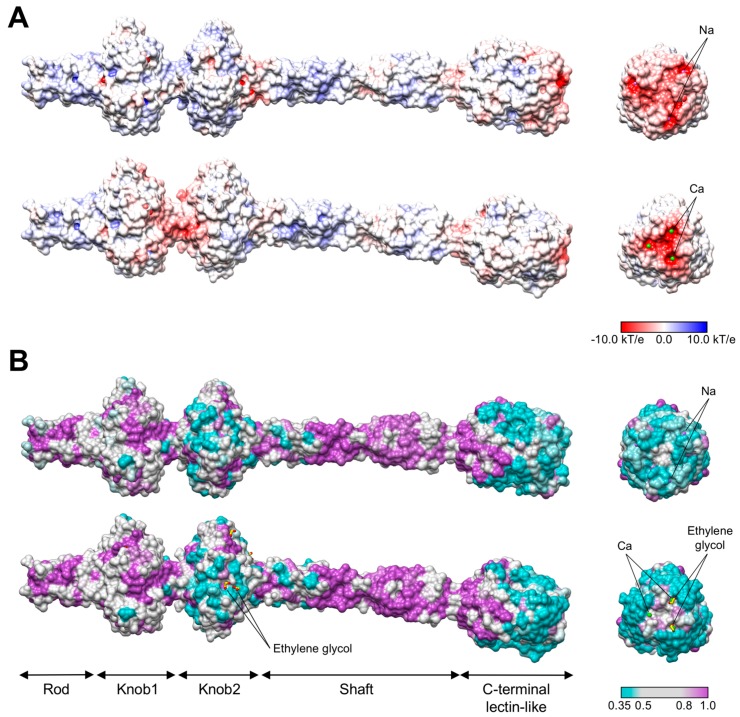
Surface properties of pyocin fibers. (**A**) Surface-mapped electrostatic potential of the R1 (top panel) and R2 (lower panel) fibers. (**B**) Sequence diversity of the surface residues of the R1 (top panel) and R2 (lower panel) fibers. The color code is given as a color bar. Panels on the left: side view. Panels on the right: C-to-N end-on view.

The greatest difference in the two structures is at the tip of the fiber because the loops connecting the β-strands have different lengths and conformations (Figure S5). The molecular surfaces of the fibers feature prominent surface grooves in this region, which might be involved in binding to the lipopolysaccharide (LPS) (Figure 6). In the R1 fiber, the grooves start at the threefold axis and extend radially outwards (Figure 6A) whereas in the R2 fiber the grooves are connected at the threefold axis, creating a “supercavity” at the tip of the fiber (Figure 6A). In both fibers, the cavity displays a strong negative charge and contains a metal-binding site—the R1 fiber contains three sodium ions, and the R2 fiber three calcium ions (Figure 3 and Figure 6, Table 2 and Table S2). The calcium cavity of R2 is located on the interface of two polypeptide chains and is much deeper than the sodium cavity of R1, which is formed by residues belonging to the same polypeptide chain. On superposition of the two fibers, the distance between the calcium and sodium ions is about 8 Å (Figure S5). The calcium ions of the R2 fiber are significantly more solvent exposed than the sodium ions of R1. The coordination polyhedra of the calcium ions are mostly complete, with some water molecules replaced by ethylene glycol molecules that diffused into crystal during cryo protection (Table 2). On the contrary, the coordination polyhedra of the sodium ions are mostly incomplete, which can be due to the poor quality of the electron density map in those regions (Table 2).

The role of the sodium and calcium ions in the structure and function of pyocin fibers is unclear at the moment. The sodium ion could be incorporated into the structure of R1 fiber during protein purification or crystallization. The calcium ion in the R2 structure takes its origins in the cell cytoplasm as the protein was never exposed to calcium containing salts during purification or crystallization. On the one hand, these ions could be important for the folding or stability of the structure. On the other hand, these ions occupy a strategic position in the fiber and might mediate binding to the cell surface LPS. Notably, a chelating agent that is used during the purification procedure does not remove the calcium ion from the R2 fiber.

A search for protein domains with folds resembling that of the C-terminal domain of the pyocin fiber using the Dali server [64] identified several structures that are trimeric and either confirmed or presumed to be able to bind and/or degrade surface polysaccharides (Table 3). The most similar structures were those of *Acinetobacter baumannii* phage AP22 tail fiber (PDB code 4MTM) and tailspike (PDB code 4Y9V), *Helix Pomatia* agglutinin (PDB code 2CGZ) [72] and Discoidin-II (PDB code 2VM9) [73] lectin-binding proteins, and another two phage receptor-binding proteins—the C-terminal domain of the phage Sf6 tailspike (PDB code 2VBK) [74] and the C-terminal domain of the *Lactococcus lactis* phage bIL170 fiber (PDB code 2FSD) [75]. None of these domains, however, display a prominent negatively charged cavity at its tip that is indicative of metal ion binding. Nevertheless, the structural similarity of the C-terminal domain of the pyocin fiber to other sugar-binding domains and phage fibers shows that it is likely involved in binding to the saccharide portion of the LPS molecule.

Based on pairwise similarity, the fiber sequences of the five previously characterized subtypes of R-pyocins can be divided into two groups—R1-like (R1 and R5) and R2-like (R2, R3, and R4) (Figure S1). As the overall folds of R1 and R2 fibers are very similar, the differences in substrate specificity must be determined by the surface properties of the proteins. We compiled a list of diverse sequences of R1-like and R2-like fibers available in the GenBank, aligned them, and mapped the degree of sequence diversity and conservation onto the structure of R1 and R2 pyocins fibers (Figure 6B). The most diverse regions are in the Knob2 domain and in the C-terminal domain. A similar trend is displayed when the two known R1-like (R1 and R5) and three R2-like fibers (R2, R3 and R4) are compared to each other, although in this case the surface of the Knob2 domain showed greater diversity than that of the C-terminal domain. Furthermore, the C-terminal domains of R2-like fibers had only one conserved amino acid substitution at the very apex of the structure: His655 of R2 and R4 was replaced with Gln655 in R3. Thus, the Knob2 domain appears to play a greater role than the C-terminal lectin-like domain in determining the spectrum of R-type pyocins. In the structure of the R2 pyocin fiber, ethylene glycol molecules that mimic various parts of the LPS bind to the Knob2 and C-terminal domains. In the latter case, the interaction of the ethylene glycol molecule with the protein involves calcium ions, further supporting the role of calcium ions in binding to the LPS (Figure 3D, Figure S5).

### 3.7. The Binding Constant of the Pyocin to the Pseudomonas Cell Surface Is Three Orders of Magnitude Greater than That of the Free Fiber

**Figure 7:**
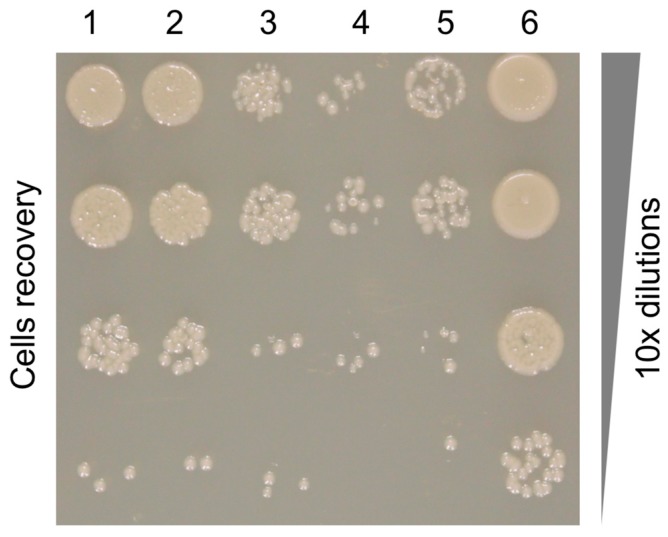
Pyocin fiber—pyocin competition assay. Recovery of viable *P. aeruginosa* 13s cells after pre-incubation with different concentrations of Ni-NTA purified fragment of R2 pyocin fiber followed by incubation with R2 pyocins. The concentration of the fiber in different columns varied as follows: 1—50 µg/mL; 2—5.0 µg/mL; 3—0.5 µg/mL; 4—0.05 µg/mL; 5—none, pyocin treatment only; 6—*P. aeruginosa* 13s cells alone. The rows are a 10-fold dilution series of the plated cells.

Earlier experiments showed that the killing spectrum of the pyocin is determined by the fibers [10,11,12]. We nevertheless decided to investigate the nature of fiber-cell surface interaction with the help of a competition experiment where binding of the pyocin particle to the cell is blocked by free fiber present in the same reaction mixture (Figure 7). We assayed the number of R2-sensitive *P. aeruginosa* 13s cells that survived coincubation with the R2 pyocin in the presence of varying concentrations of the PA0620d3 R2 fiber fragment used for structure determination (which carried the SlyD expression tag). We found that an increasing concentration of the fiber protects the cells from the pyocin. This experiment allows us to compare the interaction between the pyocin particle and its free fiber with the cell surface.

In the experimental conditions shown in Figure 7, the inhibition effect of the R2 fiber becomes apparent at a concentration of 0.5 µg/mL, which is equivalent to 2.7 nM (assuming the SlyD-fiber construct is trimeric with a molecular weight of 183 kDa) or ~10^12^ fibers per ml. This is six orders of magnitude greater than the amount of cells used in this assay (10^6^ CPU/mL) and four orders of magnitude greater than the amount of pyocin particles (10^8^ killing units per mL). In other words, there are 10^6^ fibers and 10^2^ pyocin particles (with six fibers each) per each bacterial cell in this system. Considering that the recombinant fibers and the fibers on the pyocin particle are identical and they compete for binding to the same substrate, the three orders of magnitude difference (10^6^ vs. 6 × 10^2^) in the molar amounts represents the difference in the equilibrium binding constant. The latter is likely due to a higher avidity of the pyocin to the substrate compared to that of the fiber [76]. The fiber is a trimer with several substrate binding sites, and thus is also likely to possess avidity towards the substrate. However, each pyocin particle carries six fibers that emanate from the baseplate and the binding of one fiber to the substrate promotes the binding of the others because of their spatial arrangement. The affinity of the pyocin to its substrate is therefore not a sum of the affinities of its six fibers, but is instead a cooperative non-linear function of thereof.

Notably, in this system, the fraction of the fiber that can be immobilized on the cell surface at any given time is very small. The area of the bacterial cell surface is ~4 µm^2^ or 4 × 10^8^ Å^2^. Depending on the orientation, the fiber can occupy an area of 3000 to 15,000 Å^2^ of the cell surface, which means that 10^4^–10^5^ fibers will cover the cell surface as a continuous layer. As this constitutes only 1 to 10% of the total amount of the fiber per cell present in the system, it is possible that the fibers do cover the cell surface as a continuous layer. The pyocin particle thus needs to outcompete several surface-bound fibers for successful attachment to the cell surface. The pyocin’s avidity is likely to play an important role in this process.

## 4. Discussion

**Figure 8:**
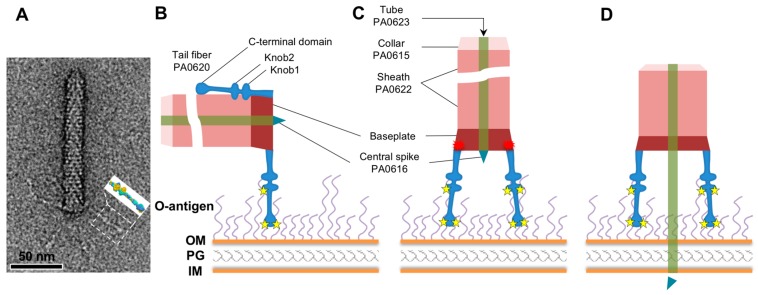
Function of the fiber in triggering sheath contraction. (**A**) A particle of R2 pyocin negatively-stained with uranyl acetate and imaged in a transmission electron microscope. The inset shows the molecular surface of the fiber fragment structure determined by X-ray crystallography. (**B**) Two spatially separated substrate-binding sites cause the fiber to bind at a certain angle relative to the cell surface. Here, the fiber is shown to be perpendicular to the cell surface, but the actual angle is determined by the structure of the substrate. (**C**) Binding of the second and subsequent fibers to the host cell surface requires their orientation relative to the cell surface to be identical or very similar to that of the first fiber. This requirement also orients the pyocin particle to be perpendicular to the cell surface. The configuration of the baseplate with all the fibers pointed towards the cell surface is unstable and causes it to initiate sheath contraction. (**D**) The sheath contracts and drives the tube through the cell envelop. The central spike protein dissociates to initiate leakage of ions from the cytoplasm. The interaction of the fibers with the bacterial surface polysaccharides is indicated with yellow stars. The fiber-to-baseplate “triggering signal” is shown with a red multipointed star. Bacterial O-antigen, outer membrane (OM), peptidoglycan layer (PG) and inner membrane (IM) are labeled.

Similar to other cell-surface binding proteins of phages, pyocin fibers are unlikely to change their structure upon cell surface binding [20,51,74,77]. At the same time, this binding initiates a cascade of structural changes that commits the phage or pyocin particle to irreversible binding to the cell (e.g., in the case of R-type pyocin—to sheath contraction). This creates an obvious paradox: The cell surface recognition signal must be transmitted to the particle along the length of fiber (about 340 Å) while the structure of the fiber changes little upon cell surface binding. Structural analysis and bioinformatics suggest Knob2 and C-terminal domain of the pyocin fibers, which are separated by a distance of about 100 Å, are likely involved in cell surface binding. This finding makes it possible to explain how the signal of cell surface binding is transmitted to the rest of the particle (Figure 8).

Because of the two sets of spatially separated ligand binding sites on the Knob2 and C-terminal domains, the fiber likely adapts a certain orientation upon interaction with the cell surface. This fiber remains bound to the cell surface while the pyocin particle is buffeted around by the surrounding solvent because of Brownian motion and/or cell swimming. Eventually, the second, third, etc. fiber binds to the surface receptor in the same or similar orientation as the first fiber. In this configuration, the pyocin particle becomes oriented perpendicular to the cell surface. At the same time, because the fibers act as rigid bodies or levers, they “unravel” the baseplate, causing it to initiate sheath contraction. We propose that this generic mechanism is employed by all contractile tail-like phages and pyocins that carry only one set of fibers (e.g., phage P2, Mu, etc.).

## Figures and Tables

**Table 1:** X-ray data collection, reduction, and refinement statistics for R2 and R1 pyocin fiber structures.

Crystal	R1 Fiber Native	R2 Fiber Native	R2 Fiber SeMet
**Data collection**			
Wavelength (Å)	1.0000	1.0000	0.9797
Number of frames	720	780	1440
Frame width (°)	0.25	0.25	0.25
Space group	P2_1_2_1_2_1_	P2_1_2_1_2_1_	P2_1_2_1_2_1_
Cell dimensions			
a, b, c (Å)	144.08, 154.46, 198.83	56.12, 126.35, 431.25	56.20, 126.82, 433.43
Resolution (Å)	50.0–2.3 (2.46–2.32)	50.0–1.9 (2.01–1.90)	50.0–2.4 (2.54–2.40)
R_meas_ (%)	10.5 (61.4)	13.0 (55.0)	7.1 (18.0)
I/σ_I_	8.8 (2.1)	8.8 (2.6)	20.8 (8.2)
CC ½ (%)	99.5 (81.0)	99.4 (81.3)	99.8(98.0)
Completeness (%)	99.2 (96.2)	99.6 (98.5)	99.3 (96.1)
Redundancy	3.4 (3.2)	3.8 (3.8)	6.7 (6.2)
Anomalous signal *	0.77 (0.65)	0.81 (0.75)	1.42 (0.78)
**Refinement**			NA **
Resolution	47.69–2.32	49.96–1.90	
No. reflections used in refinement	192,247	246,066	
No. atoms (non-H)	18,886	20,843	
Protein	16,848	16,720	
Ligand/ion	10	98	
Water	2028	4025	
R_work_/R_free_	0.169/0.206	0.160/0.208	
Average B factor (Å^2^)	60.7	24.8	
Protein	61.3	22.2	
Ligand/Ion	91.1	29.5	
Water	56.1	35.7	
R.m.s. deviations			
Bond lengths (Å)	0.003	0.009	
Bond angles (°)	0.600	0.886	
Ramachandran plot statistics			
Favored (%)	97.49	97.24	
Allowed (%)	2.51	2.76	
Outliers (%)	0.00	0.00	
Protein Data Bank accession code	6CL5	6CL6	

Statistics for the highest resolution shell are shown in the parentheses. * As defined by the program XDS [30]. ** Neither refinement nor Protein Data Bank (PDB) deposition of the SeMet derivative structure of the R2 fiber was pursued.

**Table 2:** Validation and coordination geometry analysis of metal-binding sites in R1 and R2 pyocin fiber structures with the CheckMyMetal web server [59,63]. ABC and DEF indicate the three chains of the two independent trimers comprising the asymmetric unit. The **B-factor** column gives the isotropic atomic displacement factor for the metal ion and its valence-weighted environmental average in parentheses. **Ligands** are the elemental composition of the coordination sphere. **Valence** is the overall bond valence, the average of individual bond valence values for all metal-ligand bonds. For an ideal site, the valence parameter should be the same as the charge of the metal ion. **nVECSUM** is a summation of the ligand vectors, weighted by bond valence values, and normalized by the overall valence. In a complete coordination sphere, the ligand vectors should sum to zero. The value increases as the coordination sphere becomes less complete. **Geometry** is a pattern of three-dimensional arrangement of ligands around the metal ion, as defined by the coordination chemistry: OH—Octahedral; TB—Trigonal Bipyramidal; PB—Pentagonal Bipyramidal. **gRMSD** is the overall root mean square deviation of the geometry angles (L-M-L angles) compared to the ideal geometry, in degrees. **Vacancy** is the percentage of unoccupied sites in the coordination sphere for a given geometry. The values of parameters are scored as “acceptable”, “outlier”, or “borderline” according to [59]. The outliers are bold-highlighted, and “borderline” values are underlined.

Metal Ion	B-Factor (Å2)	Ligands	Valence	nVECSUM	Geometry	gRMSD (°)	Vacancy
**R1 fiber**							
Fe^2+^ 1 (ABC)	81.0 (102.7)	N_6_	1.8	0.05	OH	5.7	0
Fe^2+^ 2 (DEF)	68.7 (79.7)	N_6_	1.9	0.10	OH	4.2	0
Mg^2+^ 3 (ABC)	153.5 (87.5)	O_6_	1.6	0.15	OH	5.8	0
Mg^2+^ 4 (DEF)	89.8 (63.9)	O_6_	1.5	0.10	OH	11.5	0
Na^+^ 5 (ABC)	65.6 (78.0)	O_5_	0.9	0.18	OH	13.0	16%
Na^+^ 6 (ABC)	75.8 (83.8)	O_5_	1.0	**0.25**	TB	13.2	0
Na^+^ 7 (ABC)	77.9 (94.7)	O_6_	0.9	0.18	OH	**23.0**	0
Na^+^ 8 (DEF)	84.6 (66.2)	O_5_	0.9	0.17	OH	14.0	16%
Na^+^ 9 (DEF)	138.4 (98.4)	O_5_	1.2	0.08	OH	16.1	16%
Na^+^ 10 (DEF)	75.5 (91.1)	O_5_	0.9	0.21	OH	17.3	16%
**R2 fiber**							
Fe^2+^ 1 (ABC)	41.6 (33.3)	N_6_	1.5	0.04	OH	4.4	0
Fe^2+^ 2 (DEF)	34.7 (35.2)	N_6_	1.6	0.11	OH	4.2	0
Mg^2+^ 3 (ABC)	23.5 (22.8)	O_6_	2.0	0.07	OH	9.0	0
Mg^2+^ 4 (DEF)	26.1 (21.2)	O_6_	1.8	0.07	OH	9.2	0
Ca^2+^ 5 (ABC)	16.7 (20.4)	O_7_	2.1	0.11	PB	11.4	0
Ca^2+^ 6 (ABC)	13.9 (16.8)	O_7_	2.2	0.11	PB	9.7	0
Ca^2+^ 7 (ABC)	15.1 (20.0)	O_7_	2.4	0.09	PB	9.9	0
Ca^2+^ 8 (DEF)	19.7 (22.9)	O_7_	1.9	0.16	PB	11.5	0
Ca^2+^ 9 (DEF)	18.0 (19.3)	O_7_	1.9	0.11	PB	10.4	0
Ca^2+^ 10 (DEF)	19.5 (21.6)	O_7_	2.0	0.11	PB	10.2	0

**Table 3:** Structural orthologs of the C-terminal domain of the R1 and R2 pyocin fibers. In all columns, the first and second number corresponds to the R1 and R2 structures, respectively. **RMSD** is the root mean square deviation of all aligned C_α_ atoms. **Lali** stands for the number of residues used in the superposition; **# res** is the number of residues in the compared structure; **% id** is the percent of sequence identity in the compared structures.

PDB Code	Z-Score	RMSD (Å)	Lali	# res	% id	Hit Description
4MTM	11.2/12.6	2.3/2.0	90/91	137	26/30	Tail fiber of bacteriophage AP22
3WMP	8.0/7.6	2.7/2.2	82/73	94	13/15	SLL-2, galactose-binding lectin
2VME	7.8/7.8	4.1/3.7	88/80	256	17/13	Discoidin-2; oligosaccharide-binding lectin
2CGZ	7.8/6.5	2.6/2.1	81/71	101	20/15	*Helix pomatia* agglutinin; oligosaccharide-binding lectin
4Y9V	6.9/6.5	3.3/3.1	87/80	603	13/13	Tailspike of bacteriophage AP22
2VBK	5.9/6.1	3.3/3.2	79/75	511	13/8	Tailspike of bacteriophage SF6
2FSD	4.7/5.5	3.2/3.0	82/83	110	10/11	Tail fiber of bacteriophage bIL170

## Supplementary Materials

The following are available online at http://www.mdpi.com/1999-4915/10/8/427/s1, Figure S1. Phylogenetic tree of fiber sequences of the five R-type pyocins. Figure S2. Crystal packing of R1 and R2 pyocin fibers. Figure S3. Location and identification of the ligand buried within the hydrophobic core of the Knob2 domain of R1 pyocin fiber. Figure S4. Location and identification of the ligand buried within the hydrophobic core of the Shaft domain of R2 pyocin fiber. Figure S5. Comparison of the structure of the C-terminal lectin-like domains of R1 and R2 fibers. Table S1. Composition of the R2 pyocin particle as determined by mass-spectrometry. Table S2. Refinement statistics and final electron density map correlation for metal ions contained in the R1 and R2 fibers. Table S3. Identification of buried compounds in the Knob2 domain of the R1 fiber and the Shaft domains of the R2 fiber.
