# Supplementary material for: Structure and Analysis of R1 and R2 Pyocin Receptor-Binding Fibers
Source: Viruses. 2018 Aug 14;10(8):427. doi: 10.3390/v10080427 (PMC6116203; doi:10.3390/v10080427)
Supplement: Supplementary file 1 [file viruses-10-00427-s001.pdf]

Supplementary Information

Structure and analysis of R1 and R2 pyocin receptor-binding fibers

Sergey A. Buth[a,*], Mikhail M. Shneider[a,b], Dean Scholl[c], Petr G. Leiman[a,*#]

This file contains:

Figures S1-S5, Tables S1-S3, and associated Figure and Table legends.

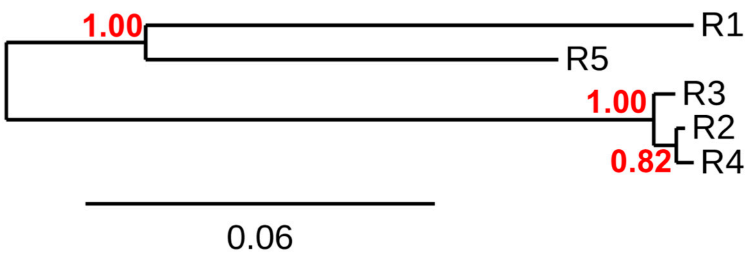

Figure S1. Phylogenetic tree of fiber sequences of the five R-type pyocins. The tree is generated with the help of the webserver Phylogeny.fr [1].

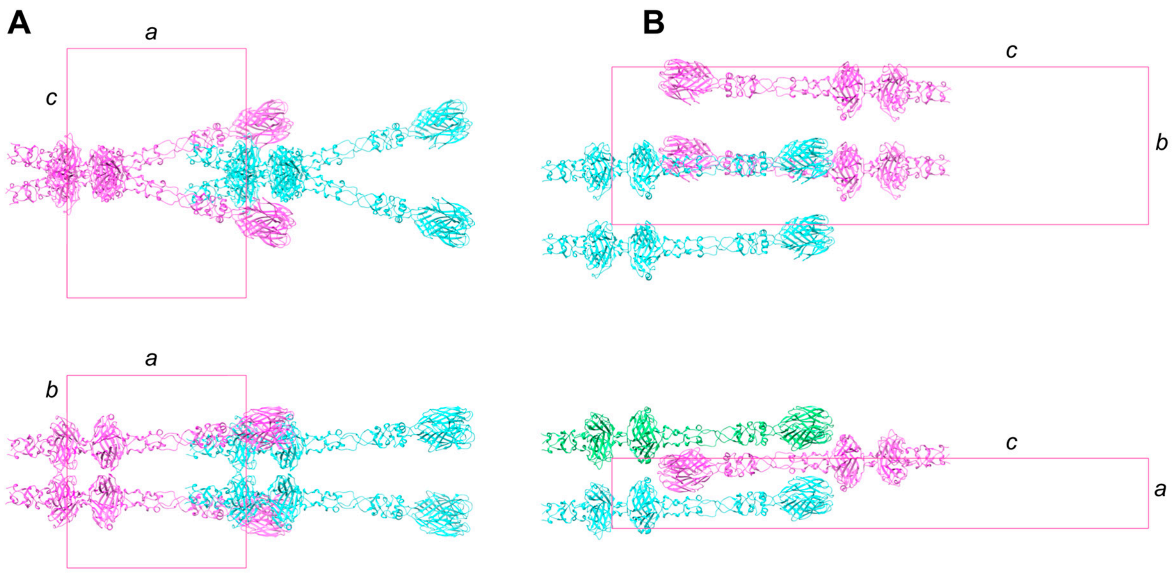

Figure S2. Crystal packing of R1 (panels A) and R2 (panels B) pyocin fibers. Two molecules comprising the asymmetric unit are shown in the same color. The unit cell is displayed in purple and cell axes are labeled. The green molecule in panel **B** demonstrates a tighter packing of the R2 fiber crystal. Despite their similar size, structure, and comparable crystallization conditions, the crystal packings of R1 and R2 fibers are markedly different. R2 fibers form interdigitated layers consisting of parallel and antiparallel units. The opposing C-terminal domains fit neatly into a space between the shaft domains. To the contrary, R1 fibers pack in a crisscross pattern with large gaps and solvent channels. The 'more logical' R2 fiber packing is nevertheless characterized by an above-average solvent content of 63%. The solvent content of the R1 fiber crystals is even higher at 74%. The tighter packing and lower solvent content are likely responsible for the higher quality diffraction data and electron density of the R2 fiber (**Table 1**).

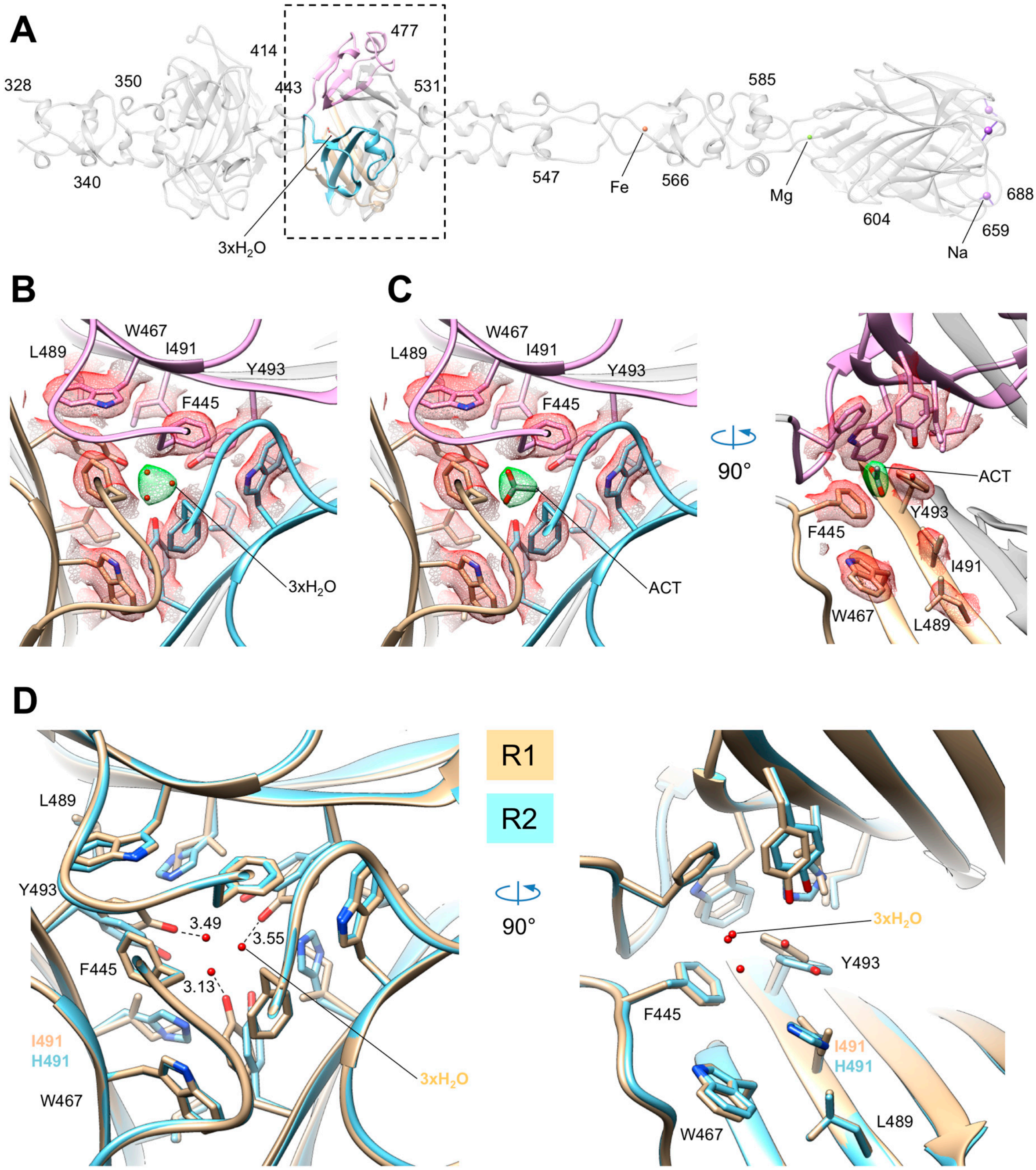

Figure S3. Location and identification of the ligand buried within the hydrophobic core of the Knob2 domain of R1 pyocin fiber. (**A**) The location of the mystery electron density in the structure of the R1 fiber Knob2 domain. The density is labeled according to its final atomic interpretation – three water molecules 3×H_2O. (**B**) An N-to-C terminus end-on view of the Knob2 domain with the three refined water molecules. The electron density of the 2Fo-Fc Fourier synthesis is contoured at 1.2 standard deviations above the mean. (**C**) An N-to-C terminus end-on view and a side cutaway view of the Knob2 domain with a refined acetate ion. (**D**) An N-to-C end-on and a side cutaway view of the superposed Knob2 domains of R1 and R2 fibers colored in tan and sky blue, respectively. Side chains of residues constituting the hydrophobic cavity that contains the ligand in question are showed and labeled. The water molecules belong to the Knob2 domain of the R1 fiber. Hydrogen bonds between the three Y493 side chains and water molecules as well as the corresponding distances are indicated.

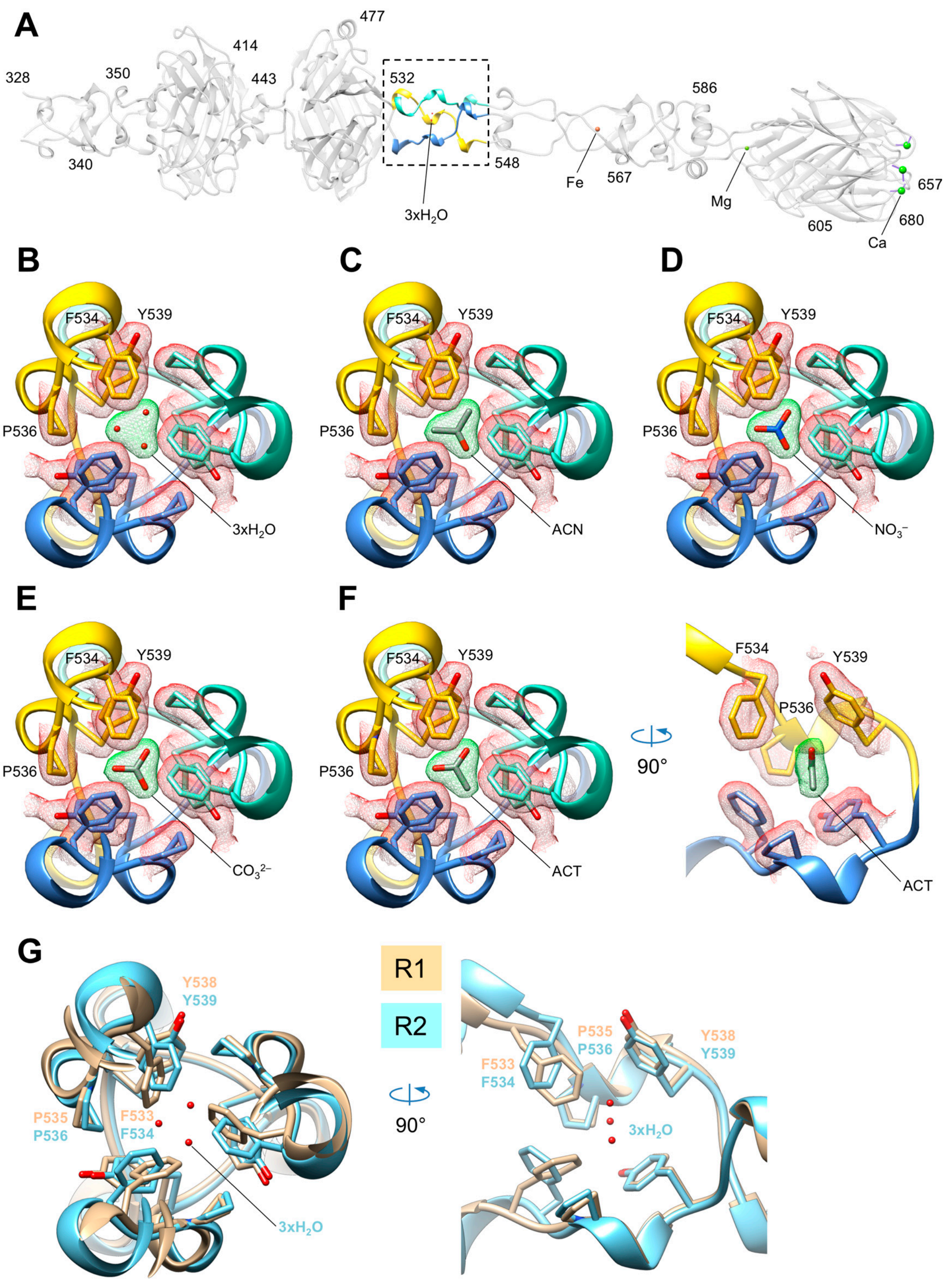

Figure S4. Location and identification of the ligand buried within the hydrophobic core of the Shaft domain of R2 pyocin fiber. (**A**) The location of the mystery electron density in the structure of the R2 fiber Shaft domain. The density is labeled according to its final atomic interpretation – three water molecules 3×H_2O. (**B**), (**C**), (**D**), (**E**), (**F** left panel) N-to-C end-on and (**F** right panel) side cutaway views of the shaft domain with different molecules fitted and refined in the density in question. ACN and ACT stand for acetone and acetate ion, respectively. The 2Fo-Fc electron density maps are contoured at 1.2 standard deviations above the mean. (**G**) An N-to-C end-on view and a side cutaway view of the superposed Shaft domains of R1 and R2 fibers colored in tan and sky blue, respectively. Side chains of residues constituting the hydrophobic cavity that contains the ligand in question are showed and labeled. The water molecules belong to the Shaft domain of the R2 fiber.

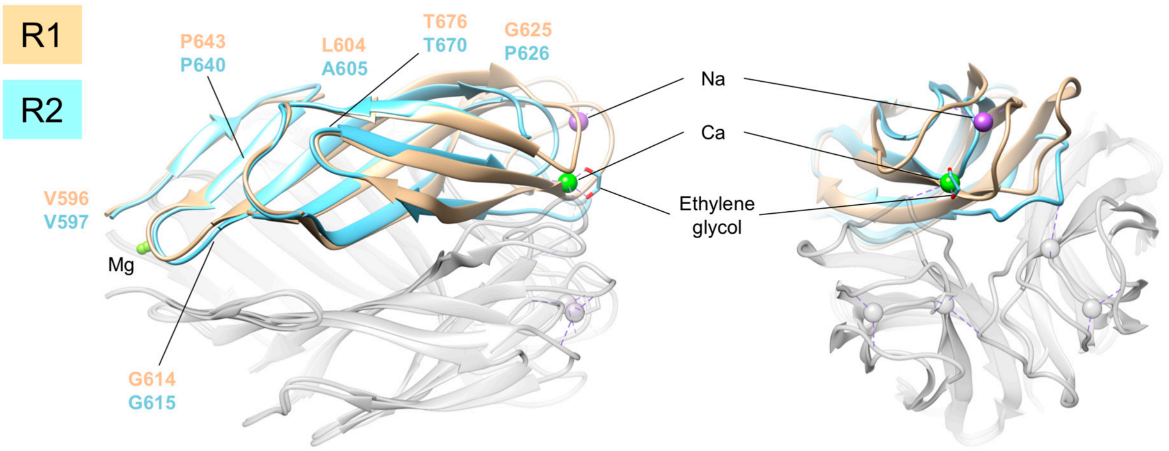

Figure S5. Comparison of the structure of the C-terminal lectin-like domains of R1 and R2 fibers. Metal ions located at the tip of the structure as well as the buried magnesium ion are shown as spheres, colored in different colors, and labeled.

Table S1. Composition of the R2 pyocin particle as determined by mass-spectrometry. The homology relationships were established with the help of the HHpred software [2] and the locations of the genes in the pyocin cluster.

Protein name in PAO1	R2 pyocin cluster nomenclature	T4 protein	Function
PA0615	Prf10	Gp15	Tail sheath terminator
PA0616	Prf11	Gp5-gp5.4	Baseplate central spike-tip complex
PA0617	Prf12	Gp25	Sheath-baseplate attachment
PA0618	Prf13	Gp6	Baseplate circularization
PA0619	Prf14	Gp7	Baseplate-tail fiber attachment
PA0620	Prf15	Gp10-gp11-gp12	Tail fiber (TF network in T4)
PA0622	Prf17	Gp18	Tail sheath
PA0623	Prf18	Gp19	Tail tube
PA0625	Prf20	Gp29	Tail length (tape measure)
PA0626	Prf21	Gp48	Centerpiece of the baseplate, interface between the hub and the tail tube
PA0627	Prf22	Gp53	Baseplate-sheath interaction and baseplate circularization, LysM domain
PA0628	Prf23	Gp27	Central hub, attaches the baseplate central spike to the tube, 3-to-6-fold symmetry adapter

Table S2. Refinement statistics and final electron density map correlation for metal ions contained in the R1 and R2 fibers. The peak heights of the 2Fo-Fc and Fc maps (given in standard deviations above the mean) were calculated with the Phenix software package [3]. CC stands for the correlation coefficient between the Fo and Fc maps. ABC and DEF indicate the three chains of the two independent trimers comprising the asymmetric unit.

**Metal ion**	B-factor (Å^2)	Occupancy	2Fo-Fc	Fc	CC
**R1 fiber**					
Fe^{2+} 1 (ABC)	81.0	1.0	8.85	8.41	0.986
Fe^{2+} 2 (DEF)	68.7	1.0	10.31	9.30	0.988
Mg^{2+} 3 (ABC)	153.5	1.0	3.30	2.64	0.970
Mg^{2+} 4 (DEF)	89.8	1.0	5.06	4.88	0.990
Na^{+} 5 (ABC)	65.6	1.0	2.86	3.66	0.956
Na^{+} 6 (ABC)	75.8	1.0	3.75	3.32	0.959
Na^{+} 7 (ABC)	77.9	1.0	2.34	3.48	0.808
Na^{+} 8 (DEF)	84.6	1.0	3.02	2.57	0.940
Na^{+} 9 (DEF)	138.4	1.0	2.70	2.19	0.944
Na^{+} 10 (DEF)	75.5	1.0	2.10	3.20	0.930
**R2 fiber**					
Fe^{2+} 1 (ABC)	41.6	1.0	10.24	10.06	0.997
Fe^{2+} 2 (DEF)	34.7	1.0	11.49	11.89	0.997
Mg^{2+} 3 (ABC)	23.5	1.0	5.07	5.09	0.985
Mg^{2+} 4 (DEF)	26.12	1.0	4.02	4.36	0.988
Ca^{2+} 5 (ABC)	16.7	1.0	10.48	11.03	0.998
Ca^{2+} 6 (ABC)	13.9	1.0	12.15	11.80	0.996
Ca^{2+} 7 (ABC)	15.1	1.0	11.04	11.64	0.998
Ca^{2+} 8 (DEF)	19.7	1.0	10.09	9.75	0.997
Ca^{2+} 9 (DEF)	18.0	1.0	10.53	9.64	0.997
Ca^{2+} 10 (DEF)	19.5	1.0	9.81	9.69	0.997

Table S3. Identification of buried compounds in the Knob2 domain of the R1 fiber and the Shaft domains of the R2 fiber. All parameters used are as in **Table S2**. ACT and ACN stand for Acetate ion and Acetone, respectively.

Ligand	B-factor (Å^2)	Occupancy	2Fo-Fc	Fc	CC
**R1 fiber**					
H_2O 1 (ABC)	54.0	1.0	1.68	1.44	0.976
H_2O 2 (ABC)	55.6	1.0	1.90	1.76	0.945
H_2O 3 (ABC)	61.7	1.0	1.73	1.01	0.977
H_2O 4 (DEF)	53.8	1.0	2.18	1.17	0.975
H_2O 5 (DEF)	50.3	1.0	2.59	1.71	0.980
H_2O 6 (DEF)	49.1	1.0	2.60	2.00	0.987
ACT 1 (ABC)	65.0	1.0	1.69	1.68	0.939
ACT 2 (DEF)	59.9	1.0	2.42	1.90	0.951
**R2 fiber**					
H_2O 1 (ABC)	30.4	1.0	2.68	2.48	0.966
H_2O 2 (ABC)	33.5	1.0	2.49	2.15	0.967
H_2O 3 (ABC)	32.9	1.0	2.68	2.35	0.976
H_2O 4 (DEF)	29.4	1.0	2.85	2.64	0.971
H_2O 5 (DEF)	40.1	1.0	1.76	1.68	0.959
H_2O 6 (DEF)	37.1	1.0	2.07	1.86	0.969
ACN 1 (ABC)	30.7	1.0	2.73	2.69	0.954
ACN 2 (DEF)	37.1	1.0	2.31	2.33	0.947
NO_3 1 (ABC)	36.1	1.0	2.91	3.13	0.971
NO_3 2 (DEF)	38.3	1.0	2.50	2.94	0.953
CO_3 1 (ABC)	35.9	1.0	2.83	2.83	0.971
CO_3 2 (DEF)	38.8	1.0	2.42	2.62	0.952
ACT 1 (ABC)	38.5	1.0	2.56	2.49	0.965
ACT 2 (DEF)	48.7	1.0	2.09	1.92	0.944

References

1. Dereeper, A.; Guignon, V.; Blanc, G.; Audic, S.; Buffet, S.; Chevenet, F.; Dufayard, J. F.; Guindon, S.; Lefort, V.; Lescot, M.; Claverie, J. M.; Gascuel, O., Phylogeny.fr: robust phylogenetic analysis for the non-specialist. *Nucleic Acids Res* **2008,** 36, (Web Server issue), W465-9.
2. Alva, V.; Nam, S. Z.; Soding, J.; Lupas, A. N., The MPI bioinformatics Toolkit as an integrative platform for advanced protein sequence and structure analysis. *Nucleic Acids Res* **2016,** 44, (W1), W410-5.
3. Adams, P. D.; Afonine, P. V.; Bunkoczi, G.; Chen, V. B.; Davis, I. W.; Echols, N.; Headd, J. J.; Hung, L. W.; Kapral, G. J.; Grosse-Kunstleve, R. W.; McCoy, A. J.; Moriarty, N. W.; Oeffner, R.; Read, R. J.; Richardson, D. C.; Richardson, J. S.; Terwilliger, T. C.; Zwart, P. H., PHENIX: a comprehensive Python-based system for macromolecular structure solution. *Acta Crystallogr D Biol Crystallogr* **2010,** 66, (Pt 2), 213-21.
